# Rare Variants Analyses Suggest Novel Cleft Genes in the African Population

**DOI:** 10.21203/rs.3.rs-3921355/v1

**Published:** 2024-02-27

**Authors:** Azeez Alade, Peter Mossey, Waheed Awotoye, Tamara Busch, Abimbola Oladayo, Emmanuel Aladenika, Mojisola Olujitan, J.J Lord Gowans, Mekonen A. Eshete, Wasiu L. Adeyemo, Erliang Zeng, Eric Otterloo, Michael O’Rorke, Adebowale Adeyemo, Jeffrey C. Murray, Justin Cotney, Salil A. Lachke, Paul Romitti, Azeez Butali, Emma Wentworth, Deepti Anand, Thirona Naicker

**Affiliations:** University of Iowa; University of Dundee; University of Iowa; University of Iowa; University of Iowa; University of Iowa; University of Iowa; Komfo Anokye Teaching Hospital and Kwame Nkrumah University of Science and Technology; Addis Ababa University; University of Lagos; University of Iowa; University of Iowa; University of Iowa; National Human Genomic Research Institute; University of Iowa; University of Connecticut; University of Delaware; University of Iowa; University of Iowa; University of Connecticut; University of Delaware; University of KwaZulu-Natal

**Keywords:** Craniofacial, Rare variants, Genetics, Transcriptomics, Orofacial clefts, Nonsyndromic

## Abstract

Non-syndromic orofacial clefts (NSOFCs) are common birth defects with a complex etiology. While over 60 common risk loci have been identified, they explain only a small proportion of the heritability for NSOFC. Rare variants have been implicated in the missing heritability. Thus, our study aimed to identify genes enriched with nonsynonymous rare coding variants associated with NSOFCs. Our sample included 814 non-syndromic cleft lip with or without palate (NSCL/P), 205 non-syndromic cleft palate only (NSCPO), and 2150 unrelated control children from Nigeria, Ghana, and Ethiopia. We conducted a gene-based analysis separately for each phenotype using three rare-variants collapsing models: (1) protein-altering (PA), (2) missense variants only (MO); and (3) loss of function variants only (LOFO). Subsequently, we utilized relevant transcriptomics data to evaluate associated gene expression and examined their mutation constraint using the gnomeAD database. In total, 13 genes showed suggestive associations (p = E-04). Among them, eight genes (*ABCB1*, *ALKBH8*, *CENPF*, *CSAD*, *EXPH5*, *PDZD8*, *SLC16A9*, and *TTC28*) were consistently expressed in relevant mouse and human craniofacial tissues during the formation of the face, and three genes (*ABCB1*, *TTC28*, and *PDZD8*) showed statistically significant mutation constraint. These findings underscore the role of rare variants in identifying candidate genes for NSOFCs.

## INTRODUCTION

Nonsyndromic orofacial clefts (NSOFCs) constitute the largest proportion of orofacial clefts (OFCs) and are estimated to affect 1.25 in every 1000 live births worldwide^1^. Due to distinct embryological origins and epidemiological patterns, NSOFCs can be broadly classified into nonsyndromic cleft lip with or without palate (NSCL/P) and nonsyndromic cleft palate only (NCSPO)^2^. The primary treatment for NSOFCs is surgical to correct structural defects. However, restoring optimal function in affected children requires a multidisciplinary team of orthodontists, maxillofacial surgeons, prosthodontists, otolaryngologists, geneticists, and pediatricians, among others^3^. In the United States, the annual cost of hospital stays for children with OFCs was over 400 million in 2013^4^. Moreover, the team of experts required for OFC care is often unavailable in resource-limited settings, leading to significant inequalities in cleft management^5^

Developing preventive or improved therapeutic strategies for NSOFCs requires a thorough understanding of their etiology. As with many complex traits, the etiology of NSOFCs is multifactorial, with genetic factors playing a considerable role^6^. According to a recent review, over 60 (> 40 associated with NSCL/P) risk loci have been implicated mainly through common variant association studies^7^. However, all identified loci/genes are estimated to explain only a small fraction (~ 25% for NSCL/P and even less for NSCPO) of the estimated heritability^8^. Low frequency/rare variants, gene-gene interactions, and gene-environmental interaction effects may likely explain the missing heritability^9^.

The role of rare variants in complex traits is well documented^10^, and a recent study found rare variants to be responsible for a larger proportion of the missing heritability in complex traits etiology^11^. However, studies evaluating the role of rare variants for NSOFCs have been restricted largely to the resequencing of known cleft candidate genes, limiting the discovery of novel genes. Although resequencing has provided insights into the burden of rare variants in these candidate genes, the small effect sizes often estimated for complex traits, together with the low MAF of rare variants (minor allele frequency [MAF] < = 0.01) makes the conventional single variants association analysis underpowered.^12–14^. A more efficient approach to rare variant analysis is to select a fixed MAF threshold and conduct an aggregate test on all variants with a MAF below this threshold within a specified region (e.g. gene), either by assigning equal weight to all variants identified or by varying weights based on the estimated variance of each variant under the null hypothesis of no association^13^. This approach has been applied successfully in discovering genes associated with complex traits like blood pressure, myocardial infarction, and schizophrenia^15–17^.

Attempts at leveraging rare variant aggregation to identify novel genes for NSOFCs are relatively new and have been limited to samples of individuals of European ancestry^8,18,19^. Additionally, these studies included all the identified rare variants within a gene, an approach that has been shown to be less sensitive due to the inclusion of synonymous variants, which often do not contribute to disease etiology^20^. To address these limitations in previous studies, we conducted gene-based rare variant aggregation tests, including only the protein-altering rare variants using our African genome-wide association study (GWAS) data. We hypothesized that genes enriched for rare protein-altering variants associated with NSOFCs would contribute to the etiology of NSOFCs. Further, we utilized transcriptomics data to provide additional evidence for the associated genes. The African population, the ancestral origin of modern humans, harbors the greatest number of genetic variations and, thus, provides a considerable opportunity for genetic discoveries^21^.

## RESULTS

### Gene-Based Rare Variant Results

Our analysis included 21, 829 rare variants (21, 333 missense variants, and 70 loss of function variant) (supplementary Table 2). We identified thirteen genes with suggestive associations (E-04 for the PA, Bonferroni corrected p-value = 0.05/5784(9E-06), E-04 for the MO, Bonferroni corrected p-value = 0.05/5676 (9E-06), and 0.05 for the LOFO model, Bonferroni corrected p-value = 0.05/26(2E-03)) were identified in the African GWAS data. Among the genes showing suggestive associations in the protein-altering (PA) model, *ABCB1* and *TTC28* were associated with NSCL/P, while *TTC12*, *PDZD8*, *FCRL4*, *CENPF*, and *SLC16A9* were associated with NSCPO. In the NSCL/P sub-group analyses into NSCLO and NSCLP, *MASP2* and *OR5K1* genes were associated with NSCLO, while *EXPH5*, *CSAD*, *ALKBH8*, and *RGL4* were associated with NSCLP. The results were similar for the missense-only (MO) model, with the addition of *ABCB1* identified with NSCL (Table 1). None of the genes showed association in the loss-of-function-only (LOFO) models. Furthermore, among the associated genes, three genes (*ABCB1*, *TTC28* and *PDZD8* genes) showed significant mutation constraint to missense mutations using the GnomeAD database.

Gene prioritization using transcriptomics data.

10 genes (*ABCB1*, *TTC28*, *TTC12*, *CSAD*, *EXPH5*, *SLC16A9*, *MASP2*, *ALKBH8*, *CENPF* and *PDZD8*) of the 13 genes showed expression during the formation of the human face. Most of the genes were biased towards some mesenchymal cells subtype except for the *PDZD8* and *ABCB1* genes (Fig. 2 and Supplementary Fig. 1). Nine of the 13 genes had mouse orthologs (*ALKBH8*, *CENPF*, *CSAD*, *EXPH5*, *MASP2*, *PDZD8*, *SLC16A9*, *TTC12*, and *TTC28*) and were analyzed using the SysFACE gene expression analysis tool. Seven of the Nine genes (*ALKBH8*, *CENPF*, *CSAD*, *EXPH5*, *PDZD8*, *SLC16A9*, and *TTC28*) showed consistently high expression and enrichment in relevant mouse craniofacial tissues – maxillary, medial and lateral eminence, and palate) (Fig. 3 and supplementary Fig. 2). Interestingly, these seven genes were also among the 10 genes that showed expression during human face development (Fig. 2).

## DISCUSSION

We conducted gene-based rare variant aggregation tests to identify novel candidate genes that could explain the missing heritability for NSOFCs. In total, we identified 13 genes with suggestive associations primarily driven by rare missense variations. Seven genes (*ALKBH8*, *CENPF*, *CSAD*, *EXPH5*, *PDZD8*, *SLC16A9*, and *TTC28*) showed consistent expression in relevant mouse and human craniofacial tissues during the formation of the face and one gene (*ABCB1*) without a mouse ortholog showed expression in human craniofacial tissues. Further, three genes (*ABCB1*, *TTC28*, and *PDZD8*) were predicted to be intolerant to missense variations.

Using biological plausibility to prioritize loci/genes with suggestive association in GWAS has been previously shown as a valid approach to bypassing the large sample size requirement needed to achieve significant association^22^. While gene mutation constraint is a good metric for identifying pathogenic genes, it is more commonly seen with a dominant disease-causing gene and may not be informative in other disease models (e.g., recessive). Thus, we prioritized the 8 associated genes (ABCB1, *ALKBH8*, *CENPF*, *CSAD*, *EXPH5*, *PDZD8*, *SLC16A9*, and *TTC28*) with consistent expression in relevant craniofacial tissues during human or mouse face development. Majority of these genes (*TTC28*, *CSAD*, *EXPH5*, *SLC16A9*, *ALKBH8*, and *CENPF*) showed biased expression towards mesenchymal cells subtypes from human craniofacial tissues. This could point to the importance of mesenchymal cells in palate formation since these genes were associated with either NSCLP or NSCPO and not NSCLO. Moreover, this could also be due to the stage (CS17) of embryonic development at which the facial prominences were harvested. The CS17 stage (~ 7 weeks post fertilization), a period that coincides with the later stages of lip formation and the beginning of palate formation.

Five genes (*ABCB1*, *TTC28*, *PDZD8*, *CENPF*, and *ALKBH8*) have been previously implicated in NSOFCs or diseases presenting with cleft phenotypes. The *ABCB1* and *TTC28* were associated with NSCL/P while the *PDZD8*, *CENPF*, *ALKBH8* genes are associated with NSCPO. These genes except the *ABCB1* without a mouse ortholog showed consistently high expression and enrichment in mouse craniofacial tissues especially the palate. The *ABCB1* gene is an ATB binding cassette gene that functions to regulate fetal exposure to xenobiotics through the placenta^23^. Single nucleotide variations in the *ABCB1* gene have been reported to increase the risk of NSCL/P^23^. The *TTC28* gene is in the 22q12.2 region and previous case report on patients with microdeletion of this region implicate this gene as potential candidate for pierre robin sequence- a condition that presents with cleft palate^24^. Furthermore, copy number variations overlapping this gene have been reported in cleft palate patients^25^. The *PDZD8* gene assists with lipid transfer from the endoplasmic reticulum to the endosomes and lysosomes^26,27^. Burden of variations though not statistically significant have been previously reported in this gene among patients with cleft lip with or without palate^28^. *CENPF* is a kinetochore associated protein that colocalizes with the *IFT88* (a ciliopathy gene) and compound heterozygous mutations in the CEPNF gene were reported in a human fetus with ciliopathic malformations, including cleft palate^29^. Mutations in *ALKBH8* cause intellectual developmental disorder, autosomal recessive 71, *MRT71* (OMIM #618504) in humans. This condition presents with craniofacial dysmorphic features, which include long lips with V-shaped upper lip, macrostomia, and retruded mandible^30^. Macrostomia and retruded mandible may cause cleft palate by impeding the elevation of palatal shelves during palate formation^31^.

We identified three potential novel genes (*CSAD*, *EXPH5*, *SLC16A9*) for NSOFCs. The burden of variants in *CSAD* and *EXPH5* were associated with NSCLP while those in the *SLC16A9* gene were associated with NSCPO. Although these genes lack previous reports of direct association with NSOFCs phenotypes, they have been implicated in processes crucial for craniofacial development. The *EXPH5* gene, for instance, has been shown to play a role in cell-cell adhesion^32^; a critical process in craniofacial morphogenesis. The *SLC16A9* gene is linked to lipid metabolic traits^33^; a functionally relevant downstream target of the non-canonical transforming growth factor beta (TGFbeta) signaling - a signaling mechanism involved in face formation. The *CSAD* gene functions in the biosynthesis of taurine and the role of taurine in organogenesis has been demonstrated in mice^34^.

In the current study, we replicated the *ABCB1* gene association which was previously reported for NSCL/P in a common variants’ association study. While some reports have demonstrated the accumulation of rare variants in genes previously identified through common variants association analyses^35^, suggesting a convergence of common and rare variants in loci/genes associated with NSOFCs phenotypes, other reports suggest that common and rare variants may act through separate loci/genes^28^. For instance, targeted sequencing of regions surrounding genome-wide significant loci for NSOFCs showed no evidence of rare variants burden in genes/regulatory regions proximal to these loci^28^. Additionally, rare variants are population-dependent, which could explain why we did not replicate previously reported genes/loci from rare variant association studies in other populations. Furthermore, our tests of association require that we exclude any gene with only one rare variant, even if the same gene harbored common variants. This might have resulted in the omission of genes with contributions from both rare and common variants if participants in our cohort only harbor one rare variant in the gene. Therefore, future studies should consider a model that allows for the incorporation of both common and rare variants.

Our study has some limitations. First, we used array-based genotype data for discovery. This approach means some rare variants were not examined. Second, we controlled for population stratification by adding the top 10 genotype PCs as covariates in our gene-based association regression models. Adjusting for top PCs has been shown to prevent *p-value* inflation and reduce false positive rates in common variant analysis, but its performance for rare variant analysis remains controversial^36^. The reason is that rare variants, being newer mutations, reflect a more granular population substructure compared to common variants^37^. Finally, we restricted our analysis to only the coding (missense and Lof) and splice-altering variants. Rare variants including insertions/deletions in non-coding regulatory regions also contribute to the etiology of NSOFCs; however, defining these regions’ analytical units and their functional characterization remains a challenge. Hence, future studies should use WGS data for the gene-based analysis to capture more rare variants and leverage annotated craniofacial enhancers regions to analyze rare variants in non-coding regions.

In summary, we identified 13 genes with suggestive associations in our GWAS data. Among the 13 genes, 3 genes (*ABCB1*, *TTC28* and *PDZD8*) were predicted to be intolerant to missense variations. Human and mouse transcriptomics data further supported the association of 8 genes. Of the 8 genes, five genes (*ABCB1*, *TTC28*, *PDZD8*, *CENPF*, *ALKBH8*) were previously associated with NSOFCs or diseases presenting with cleft phenotypes. The remaining three genes (*CSAD*, *EXPH5*, *SLC16A9*) are potentially novel candidate genes for NSOFCs.

## METHODS

### GWAS Study Participants

The details of the GWAS participants have been previously published^38^. Briefly, NSOFC case children were recruited during surgical repair at cleft clinics and free surgical missions sponsored by Smile Train in Nigeria, Ghana, and Ethiopia. The cleft surgeons at each participating site used a standardized phenotyping protocol (physical examination and clinical photographs) to confirm NSOFC status. Additionally, echocardiography was used to rule out the presence of congenital heart defects to ensure nonsyndromic status. Control children were those without a birth defect diagnosis attending immunization/welfare clinics at the same center where the case children were recruited. To be eligible to participate in the study, case and control children must have biological parents of African ancestry who reside in Africa. Our sample included 1019 NSOFC case children and 2150 unrelated control children. Among the cases, 810 had non syndromic cleft lip with or without palate (NSCL/P) – 394 non-syndromic cleft lip only (NSCLO), 420 non-syndromic cleft lip and palate (NSCLP), and 205 had non-syndromic cleft palate only (NSCPO). The distribution of the GWAS study participants by cleft status and country of origin is shown in Supplementary Table 1.

### Data Collection, DNA Extraction, and Genotyping

Demographic information (age, sex, and residential location) and limited exposure information were obtained. Saliva specimens were collected using the Oragene saliva kit, de-identified and shipped to the Butali laboratory at the University of Iowa. DNA was extracted from the saliva using the standard Oragene saliva DNA extraction protocol and quantified using Qubit (http://www.invitrogen.com/site/us/en/home/brands/Product-Brand/Qubit.html;Thermo Fisher Scientific,Grand Island, NY). As part of internal QC, Taqman XY genotyping was used for sex confirmation. Subsequently, aliquoted DNA was sent to the Center for Inherited Disease Research (Baltimore, Maryland, USA) for genotyping. The Illumina Multi-Ethnic Genotyping Array MEGA v2 15070954 A2 (genome build 37), which has over 2 million variants including over 60 000 rare variants selected from populations of African origin, was used for the genotyping. Details on genotyping and QC measures have been previously published^38^.

### Data Analysis

#### Gene-Based Analyses

**Figure 1:**
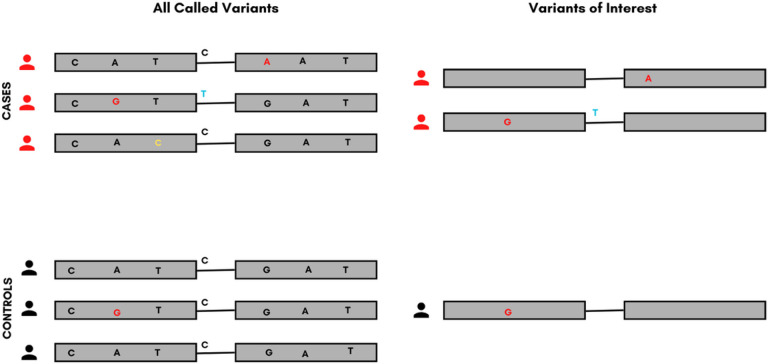
The process of sorting for variants within a gene of interest. The left side of the image shows all variants identified within the gene after sequencing. The variants of interest on the right were predicted to result in a change in amino acid or affect splicing by in-silico predictive tools and were included in our analysis. Color codes; ***Red***- Non-synonymous variants in protein-coding regions; ***Yellow***- Synonymous variants (not selected for analysis); ***Blue***- Splice-site variants.

**Figure 2:**
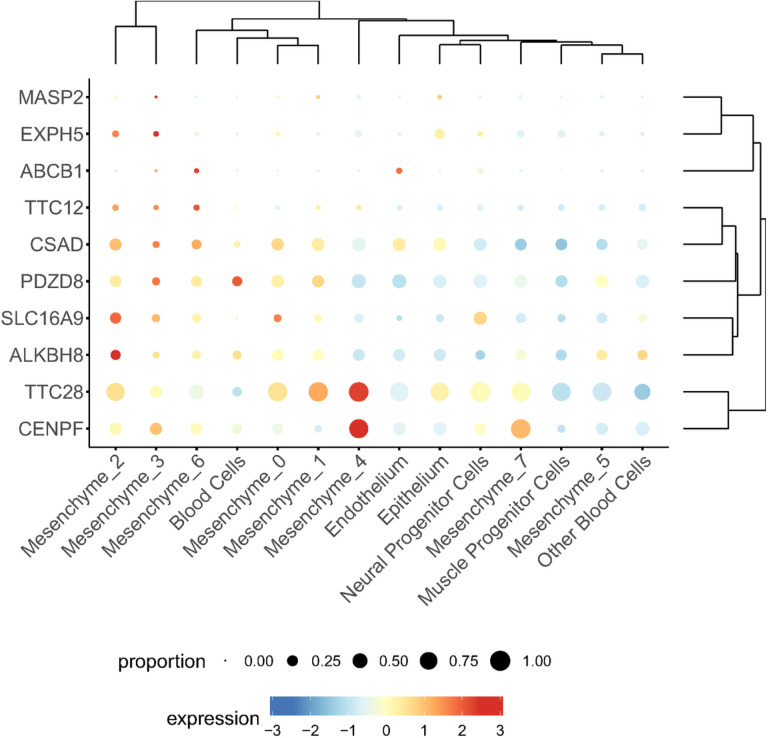
Bubble plots of gene expression for associated genes in each of the major cell types from human craniofacial tissues of CS17 embryos. Size correlates to the percent of cells per cluster which express the gene. Color corresponds to the average expression of that gene within cells of that cluster (Low = blue, high = red).

**Figure 3:**
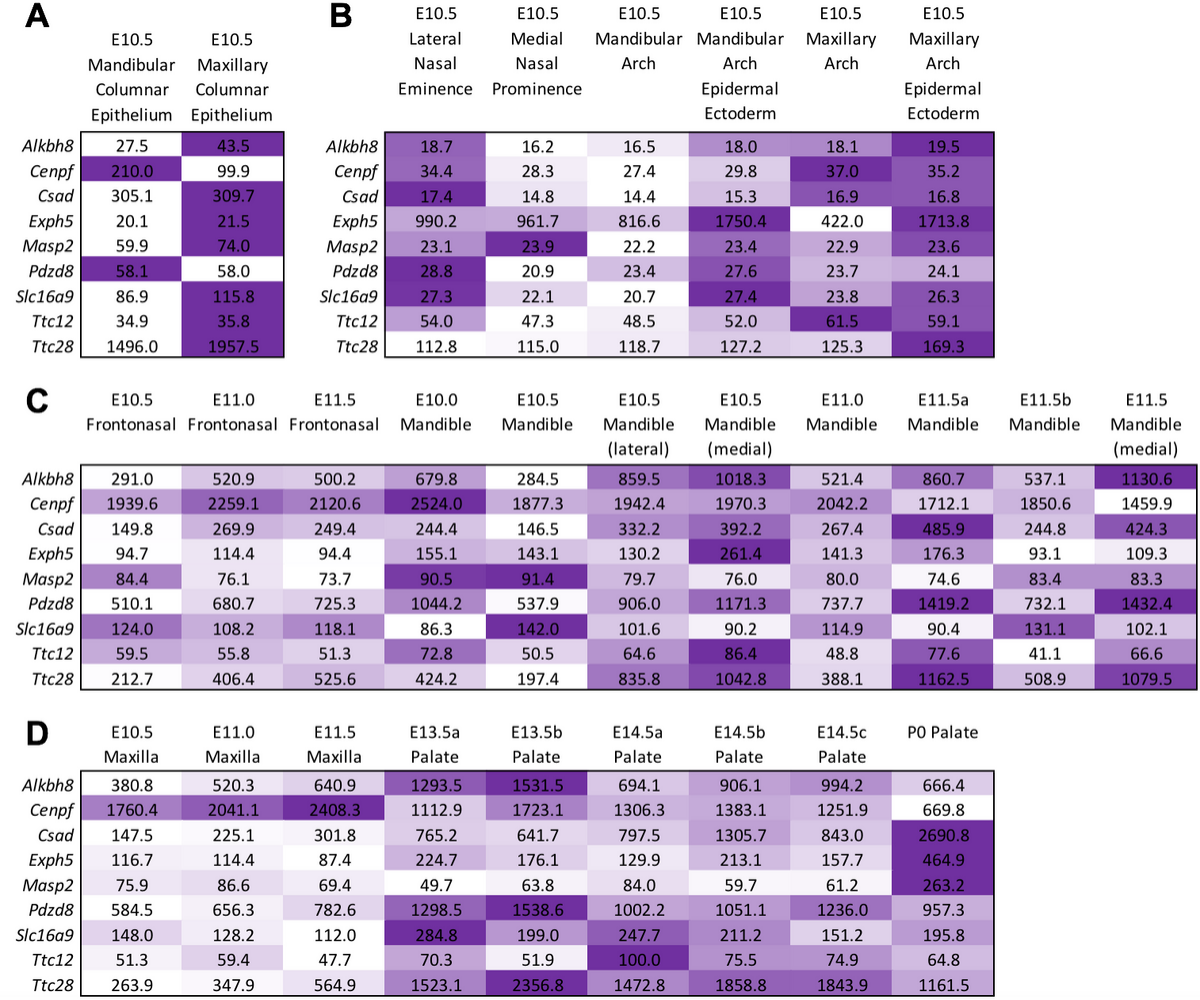
SysFACE-based expression analysis of candidate genes in mouse facial development. Mouse orthologs for 9 of the 13 human candidate genes were examined using the SysFACE tool that is based on microarray gene expression data from isolated facial tissue in mouse development. Heat-map denotes the relative expression of individual genes at various stages of mouse embryonic (E) or postnatal (P) development in specific facial tissues, namely (A) Mandibular and maxillary columnar epithelium, (B) nasal eminence/prominence, and mandibular and maxillary arch, (C) frontonasal and mandible, (D) maxilla and palate. The intensity of the color in the heat-map (row-wise) is representative of the extent of candidate gene expression based on the average fluorescence signal intensity in the specific tissue. Note that for palate, there were independent datasets for E13.5 and E14.5 and these are denoted as E13.5a, E13.5b, etc. FaceBase datasets generated on Affymetrix Mouse Gene 1.0 ST Array microarray were meta-analyzed for (A, B) and Affymetrix Mouse Genome 430 2.0 Array microarray datasets were meta-analyzed for (C, D).

Variant predication was performed using annotate variation (ANNOVAR) to identify the functional consequences (synonymous, nonsynonymous, and splice-altering) of each variant. Splice altering and non-synonymous variants (variations resulting in either amino acid change or premature termination of the protein) were filtered against the 1000 genome (1KG) population databases (http://www.1000genomes.org/) to identify rare variants (found in < = 1% of Africans included in the database). Additionally, we filtered for only the variants with a MAF < = 1% in the controls included in our sample (Supplementary table 2). Subsequently, genes with two or more rare non-synonymous variants in the data were filtered to satisfy the aggregation requirement for the proposed analyses (Fig. 1). For the analysis, both non-synonymous and splice-altering variants were included and referred to as “protein-altering”. Three gene-based collapsing models were used: (1) protein-altering (PA), (2) missense only (MO), and (3) loss of function only (LOFO). The different phenotypes (NSCL/P and NSCPO) were analyzed independently and separately. NSCL/P was further subdivided into nonsyndromic cleft lip only (NSCLO) and nonsyndromic cleft lip and palate (NSCLP). Gene-based rare variant aggregate tests were used to identify genes enriched in rare variants associated with NSOFCs. Three rare variant aggregation tests were conducted: the combined multivariate and collapsing (CMC) test, the sequence kernel association test (SKAT), and the SKAT-O test. The first two tests are complementary, operating under different assumptions^39^. The CMC test, being a burden test, assumes the same direction of effects for all the variants within a gene, whereas SKAT is a variance component test that allows for an opposing direction of effects^39^. The SKAT-O test is an omnibus test that is more robust and efficient across different scenarios^39^. In all three tests, population stratification and sex were controlled for by adding the top 18 principal components (PC) and child sex into the regression-based models. The lack of prior knowledge about the underlying biology of these variants precluded the ability to select one optimal test. Hence, the decision to conduct the three tests where the CMC and SKAT will show rigor and SKAT-O will confirm reproducibility. The Bonferroni correction was used to adjust for multiple testing and set the cutoff for statistical significance at a 5% error rate to 0.05 divided by the number of genes tested and a 10^2^ higher threshold for suggestive significance. A gene was considered significant if it showed a statistically significant or suggestive significant association in either CMC and SKATO or SKAT and SKATO. Rare variant aggregation tests were conducted on our GWAS data using the SKAT R package (https://cran.r-project.org/web/packages/SKAT/index.html) or rare variant association tests implemented under the case-control study design. Further, we used the genomeAD gene mutation constraint prediction tool (https://gnomad.broadinstitute.org/help/constraint) to identify associated genes level of intolerance to mutational changes.

#### Gene Expression Analyses

To provide biological insight, expression of the associated genes during organogenesis of the human and mouse faces was examined. The human craniofacial gene expression dataset was generated from single-nuclei RNA-seq of craniofacial prominences of human CS17 embryos. The CS17 stage corresponds to ~ 7 weeks post-fertilization which coincides with the later stage of lip formation and the early stage of palate formation. Additional details on the RNA seq data quality control and analysis was reported by Yankee et al. 2023^27^.

SysFACE analysis was performed as previously described^40^ using GSE7759, GSE22989, GSE31004, and GSE11400 microarray data (Affymetrix Mouse Genome 430 2.0 Array) and GSE55965 microarray data (Affymetrix Mouse Gene 1.0 ST Array). The datasets were analyzed using affy package in R. Multiple probesets representing individual genes were normalized and the probeset with highest median expression was considered representative of gene expression. WB data generated on Affymetrix Mouse Genome 430 2.0 Array platform as previously described was used for enriched expression analysis.

## Figures and Tables

**Table 1:** Gene-based results for the protein-altering and missense only models.

Gene	P.Burden	P.SKAT	P.SKATO	No of variants tested	Missense constraint Z score
NSCL/P - all protein altering variants					
*ABCB1*	0.0001	0.0006	0.0002	2	2.7600*
*TTC28*	0.0053	0.0004	0.0005	2	3.4500*
NSCLO - all protein altering variants					
*MASP2*	0.0007	0.0001	0.0002	2	−0.2100
*OR5K1*	0.0016	0.0006	0.0006	2	−1.0600
NSCLP-all protein altering variants					
*EXPH5*	0.0009	0.0002	0.0002	9	0.8900
*CSAD*	0.0029	0.0002	0.0003	2	0.7700
*ALKBH8*	0.0044	0.0002	0.0004	5	0.5400
*RGL4*	0.0009	0.0005	0.0007	10	0.1000
NSCPO - all protein altering variants					
*TTC12*	0.0001	0.0010	0.0003	2	0.5900
*PDZD8*	0.0001	0.0018	0.0003	3	2.5300*
*FCRL4*	0.0001	0.0017	0.0003	6	−0.1400
*CENPF*	0.2243	0.0003	0.0007	12	1.3700
*SLC16A9*	0.0035	0.0005	0.0007	2	0.8400
NSCL/P - missense only					
*TTC28*	0.0003	0.0003	0.0004	2	3.4500*
*ABCB1*	0.0006	0.0006	0.0001	2	2.7600*
NSCLO - missense only					
*MASP2*	0.0007	0.0001	0.0002	2	−0.2100
*ABCB1*	0.0002	0.5983	0.0005	2	2.7600*
*OR5K1*	0.0016	0.0006	0.0007	2	−1.0600
NSCLP missense only					
*EXPH5*	0.0009	0.0002	0.0003	9	0.8900
*CSAD*	0.0027	0.0002	0.0003	2	0.7700
*ALKBH8*	0.0045	0.0002	0.0005	5	0.5400
*RGL4*	0.0009	0.0005	0.0007	10	0.1000
NSCPO missense only					
*PDZD8*	0.0001	0.0016	0.0003	3	2.5300*
*TTC12*	0.0002	0.0012	0.0004	2	0.5900
*FCRL4*	0.0002	0.0020	0.0005	6	−0.1400
*CENPF*	0.2345	0.0003	0.0008	12	1.3700
*SLC16A9*	0.0035	0.0005	0.0009	2	0.8400

• Positive Z-scores indicate more constraint (fewer observed variants than expected), and negative scores indicate less constraint (more observed variants than expected) as observed in the genomeAD dataset.

* Statistically significant missense constraint Z score.

## Data Availability

Data available through dbGAP Accession Number: phs001090.v1. p1

## References

[1] Mossey P. A. & Modell B. Epidemiology of oral clefts 2012: an international perspective. Front Oral Biol 16, 1–18 (2012). 10.1159/000337464 22759666

[2] Calzolari E. Associated anomalies in multi-malformed infants with cleft lip and palate: An epidemiologic study of nearly 6 million births in 23 EUROCAT registries. American journal of medical genetics Part A 143, 528–537 (2007). 10.1002/ajmg.a.31447 17286264

[3] Banerjee M. & Dhakar A. S. Epidemiology-clinical profile of cleft lip and palate among children in India and its surgical consideration. CJS 2, 45–51 (2013).

[4] Arth A. C., Tinker S. C., Simeone R. M., Ailes E. C., Cragan J. D. & Grosse S. D. Inpatient Hospitalization Costs Associated with Birth Defects Among Persons of All Ages - United States, 2013. MMWR Morb Mortal Wkly Rep 66, 41–46 (2017). 10.15585/mmwr.mm6602a1 28103210 PMC5657658

[5] Nicholas D. S., Jean Calleja A., Gareth D., Felicity V. M., Peter H. & Martin P. Equality in cleft and craniofacial care. Equality in cleft and craniofacial care 7, 35 (2020). 10.20517/2347-9264.2020.99

[6] Beaty T. H., Marazita M. L. & Leslie E. J. Genetic factors influencing risk to orofacial clefts: today’s challenges and tomorrow’s opportunities. F1000Res 5, 2800 (2016). 10.12688/f1000research.9503.1 27990279 PMC5133690

[7] Alade A., Awotoye W. & Butali A. Genetic and epigenetic studies in non-syndromic oral clefts. Oral Dis 28, 1339–1350 (2022). 10.1111/odi.14146 35122708

[8] Leslie E. J. Association studies of low-frequency coding variants in nonsyndromic cleft lip with or without cleft palate. Am J Med Genet A 173, 1531–1538 (2017). 10.1002/ajmg.a.38210 28425186 PMC5444956

[9] Génin E. Missing heritability of complex diseases: case solved? Hum Genet 139, 103–113 (2020). 10.1007/s00439-019-02034-4 31165258

[10] Momozawa Y. & Mizukami K. Unique roles of rare variants in the genetics of complex diseases in humans. J Hum Genet 66, 11–23 (2021). 10.1038/s10038-020-00845-2 32948841 PMC7728599

[11] Wainschtein P. Recovery of trait heritability from whole genome sequence data. bioRxiv, 588020 (2021). 10.1101/588020

[12] Gorlov I. P., Gorlova O. Y., Sunyaev S. R., Spitz M. R. & Amos C. I. Shifting paradigm of association studies: value of rare single-nucleotide polymorphisms. Am J Hum Genet 82, 100–112 (2008). 10.1016/j.ajhg.2007.09.006 18179889 PMC2253956

[13] Lee S., Abecasis G. R., Boehnke M. & Lin X. Rare-variant association analysis: study designs and statistical tests. Am J Hum Genet 95, 5–23 (2014). 10.1016/j.ajhg.2014.06.009 24995866 PMC4085641

[14] Moutsianas L. Class II HLA interactions modulate genetic risk for multiple sclerosis. Nat Genet 47, 1107–1113 (2015). 10.1038/ng.3395 26343388 PMC4874245

[15] Do R., Balick D., Li H., Adzhubei I., Sunyaev S. & Reich D. No evidence that selection has been less effective at removing deleterious mutations in Europeans than in Africans. Nature Genetics 47, 126–131 (2015). 10.1038/ng.3186 25581429 PMC4310772

[16] Genovese G. Increased burden of ultra-rare protein-altering variants among 4,877 individuals with schizophrenia. Nat Neurosci 19, 1433–1441 (2016). 10.1038/nn.4402 27694994 PMC5104192

[17] Liu C. Meta-analysis identifies common and rare variants influencing blood pressure and overlapping with metabolic trait loci. Nat Genet 48, 1162–1170 (2016). 10.1038/ng.3660 27618448 PMC5320952

[18] Shaffer J. R. Association of low-frequency genetic variants in regulatory regions with nonsyndromic orofacial clefts. American journal of medical genetics. Part A 179, 467–474 (2019). 10.1002/ajmg.a.61002 30582786 PMC6374160

[19] Curtis S. W. Rare genetic variants in SEC24D modify orofacial cleft phenotypes. medRxiv (2023). 10.1101/2023.03.24.23287714

[20] Li B. & Leal S. M. Methods for detecting associations with rare variants for common diseases: application to analysis of sequence data. Am J Hum Genet 83, 311–321 (2008). 10.1016/j.ajhg.2008.06.024 18691683 PMC2842185

[21] Conrad D. F. A worldwide survey of haplotype variation and linkage disequilibrium in the human genome. Nat Genet 38, 1251–1260 (2006). 10.1038/ng1911 17057719

[22] Hammond R. K. Biological constraints on GWAS SNPs at suggestive significance thresholds reveal additional BMI loci. eLife 10, e62206 (2021). 10.7554/eLife.62206 33459256 PMC7815306

[23] Omoumi A. Fetal polymorphisms at the ABCB1-transporter gene locus are associated with susceptibility to non-syndromic oral cleft malformations. Eur J Hum Genet 21, 1436–1441 (2013). 10.1038/ejhg.2013.25 23443032 PMC3831066

[24] Davidson T. B. Microdeletion del(22)(q12.2) encompassing the facial development-associated gene, MN1 (meningioma 1) in a child with Pierre-Robin sequence (including cleft palate) and neurofibromatosis 2 (NF2): a case report and review of the literature. BMC Medical Genetics 13, 19 (2012). 10.1186/1471-2350-13-19 22436304 PMC3359208

[25] Conte F., Oti M., Dixon J., Carels C. E., Rubini M. & Zhou H. Systematic analysis of copy number variants of a large cohort of orofacial cleft patients identifies candidate genes for orofacial clefts. Hum Genet 135, 41–59 (2016). 10.1007/s00439-015-1606-x 26561393 PMC4698300

[26] Al-Amri A. H. *PDZD8* Disruption Causes Cognitive Impairment in Humans, Mice, and Fruit Flies. Biological Psychiatry 92, 323–334 (2022). 10.1016/j.biopsych.2021.12.017 35227461 PMC9302898

[27] Yankee T. N. Integrative analysis of transcriptome dynamics during human craniofacial development identifies candidate disease genes. Nature Communications 14, 4623 (2023). 10.1038/s41467-023-40363-1 PMC10397224 37532691

[28] Leslie E. J. Identification of functional variants for cleft lip with or without cleft palate in or near PAX7, FGFR2, and NOG by targeted sequencing of GWAS loci. Am J Hum Genet 96, 397–411 (2015). 10.1016/j.ajhg.2015.01.004 25704602 PMC4375420

[29] Waters A. M. The kinetochore protein, CENPF, is mutated in human ciliopathy and microcephaly phenotypes. J Med Genet 52, 147–156 (2015). 10.1136/jmedgenet-2014-102691 25564561 PMC4345935

[30] Saad A. K. Neurodevelopmental disorder in an Egyptian family with a biallelic ALKBH8 variant. Am J Med Genet A 185, 1288–1293 (2021). 10.1002/ajmg.a.62100 33544954 PMC8450764

[31] Diewert V. M. Correlation between mandibular retrognathia and induction of cleft palate with 6-aminonicotinamide in the rat. Teratology 19, 213–227 (1979). 10.1002/tera.1420190212 157558

[32] Bare Y., Chan G. K., Hayday T., McGrath J. A. & Parsons M. Slac2-b Coordinates Extracellular Vesicle Secretion to Regulate Keratinocyte Adhesion and Migration. J Invest Dermatol 141, 523–532.e522 (2021). 10.1016/j.jid.2020.08.011 32890627

[33] Ren T., Jones R. S. & Morris M. E. Untargeted metabolomics identifies the potential role of monocarboxylate transporter 6 (MCT6/SLC16A5) in lipid and amino acid metabolism pathways. Pharmacol Res Perspect 10, e00944 (2022). 10.1002/prp2.944 35466588 PMC9035569

[34] Park E., Park S. Y., Dobkin C. & Schuller-Levis G. Development of a Novel Cysteine Sulfinic Acid Decarboxylase Knockout Mouse: Dietary Taurine Reduces Neonatal Mortality. Journal of Amino Acids 2014, 346809 (2014). 10.1155/2014/346809 24639894 PMC3929995

[35] Leslie E. J. & Murray J. C. Evaluating rare coding variants as contributing causes to non-syndromic cleft lip and palate. Clin Genet 84, 496–500 (2013). 10.1111/cge.12018 22978696 PMC3788862

[36] Chen W., Coombes B. J. & Larson N. B. Recent advances and challenges of rare variant association analysis in the biobank sequencing era. Frontiers in Genetics 13 (2022). 10.3389/fgene.2022.1014947 PMC9582646 36276986

[37] O’Connor T. D. Rare Variation Facilitates Inferences of Fine-Scale Population Structure in Humans. Molecular Biology and Evolution 32, 653–660 (2014). 10.1093/molbev/msu326 25415970 PMC4327153

[38] Butali A. Genomic analyses in African populations identify novel risk loci for cleft palate. Hum Mol Genet 28, 1038–1051 (2019). 10.1093/hmg/ddy402 30452639 PMC6400042

[39] Lee S. H., Yang J., Goddard M. E., Visscher P. M. & Wray N. R. Estimation of pleiotropy between complex diseases using single-nucleotide polymorphism-derived genomic relationships and restricted maximum likelihood. Bioinformatics 28, 2540–2542 (2012). 10.1093/bioinformatics/bts474 22843982 PMC3463125

[40] Awotoye W. Genome-wide Gene-by-Sex Interaction Studies Identify Novel Nonsyndromic Orofacial Clefts Risk Locus. J Dent Res 101, 465–472 (2022). 10.1177/00220345211046614 34689653 PMC8935575

